# Robotic Duodenojejunostomy for a Rare Case of Acute Idiopathic Superior Mesenteric Artery Syndrome in a Teenage Girl

**DOI:** 10.1055/a-2662-2517

**Published:** 2025-08-05

**Authors:** Thibault Planchamp, Olivier Abbo

**Affiliations:** 1 Department of Pediatric Surgery, Hôpital des Enfants, CHU Toulouse, Toulouse University, Toulouse, Occitanie, France

**Keywords:** superior mesenteric artery syndrome, robotic surgery, pediatric

## Abstract

Superior mesenteric artery (SMA) syndrome is a rare cause of proximal bowel obstruction in pediatric surgery. We present the management of a rare, idiopathic, and acute form of SMA syndrome in a teenage girl, successfully treated with a robotic approach. A 14.5-year-old girl with no prior medical history and a normal body mass index (BMI) for her age (18.4 kg/m ^2^ ) was admitted to our department with acute proximal bowel obstruction syndrome. Initial imaging, including an abdominal X-ray, computed tomography scan, and upper gastrointestinal tract radiography, confirmed the diagnosis of SMA syndrome. Conservative management was initiated with nasogastric tube placement, postural adjustments, and optimal nutritional support. However, symptoms persisted, and surgery was performed 5 months after the initiation of conservative treatment. A robotic-assisted duodenojejunostomy, preserving the fourth portion of the duodenum, was performed without intraoperative complications. Postoperatively, the patient experienced immediate symptom relief and was discharged on postoperative day 4. The postoperative course and long-term follow-up (7 years) were uneventful. This case report describes an acute, idiopathic form of SMA syndrome successfully treated with robotic-assisted duodenojejunostomy in a teenage girl with a normal BMI. To our knowledge, this is only the second reported case of robotic surgery for SMA syndrome in the pediatric literature.

## Introduction

Superior mesenteric artery (SMA) syndrome, also known as Wilkie's syndrome, is a rare cause of mechanical obstruction of the proximal small bowel. 1 2

It results from the compression of the third portion of the duodenum between the SMA and the abdominal aorta due to congenital or acquired risk factors. Typically observed in female patients with a low body mass index (BMI), an insidious onset of symptoms occurs in 90% of cases.

However, SMA syndrome may be idiopathic in 40% of cases and can rarely present in an acute form.

Conservative treatment remains the first-line therapy, but surgery becomes necessary in cases of treatment failure. Several surgical techniques have already been described, but the advent of robotic surgery offers new treatment possibilities.

Here, we present a rare form of idiopathic and acute SMA syndrome in a teenage girl, successfully treated with a robotic approach.

## Case Presentation

A 14.5-year-old girl with no prior medical history and a normal BMI for age (18.4 kg/m ^2^ ; weight: 41.3 kg; height: 150 cm) was admitted to our department for acute proximal bowel obstruction syndrome with abdominal pain, epigastric distension, and bilious emesis. Symptoms had begun the day before.

**Fig. 1:**
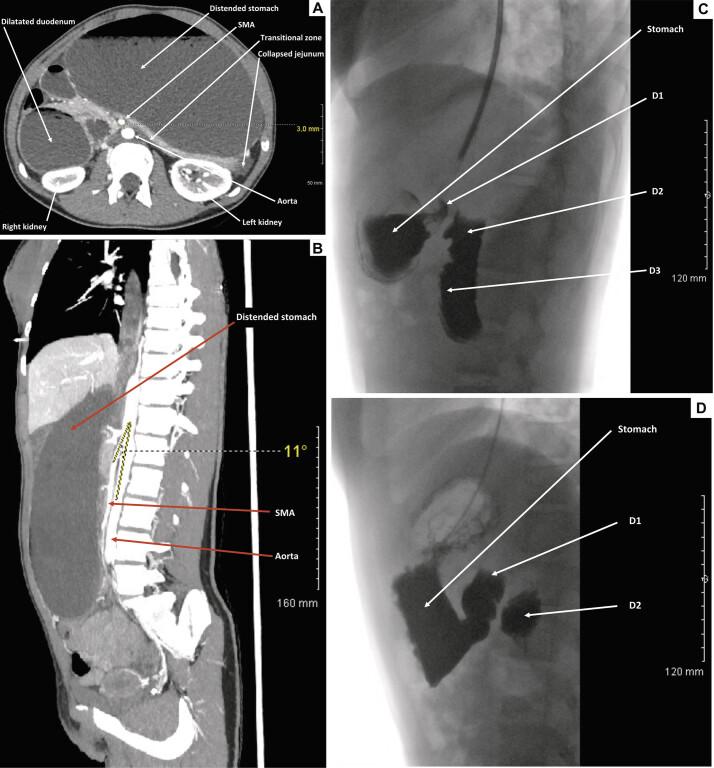
( **A** ) Axial section of an abdominal CT scan with contrast injection at the arterial phase, showing significant gastric dilation and moderate proximal duodenal dilation. The aortomesenteric distance is reduced to 3 mm. ( **B** ) Sagittal section of an abdominal CT scan with contrast injection at the arterial phase, showing significant gastric dilation. The aortomesenteric angle is reduced to 11 degrees. ( **C** ) Lateral view of an upper gastrointestinal tract radiography performed on the day of the emergency consultation, showing significant gastric dilation and moderate dilation of D1, D2, and D3. ( **D** ) Lateral view of an upper gastrointestinal tract radiography performed three weeks after the initiation of medical management, showing a reduction in gastric and duodenal dilation, although some dilation persisted. D1, first portion of the duodenum; D2, second portion of the duodenum; D3, third portion of the duodenum; SMA, superior mesenteric artery.

An abdominal computed tomography (CT) scan with intravenous contrast revealed SMA syndrome, characterized by an aortomesenteric distance of 3 mm ( Fig. 1A ) and an aortomesenteric angle of 11 degrees ( Fig. 1B ). Significant gastric dilation and moderate proximal duodenal dilation were observed ( Fig. 1A–C ). The diagnosis was confirmed with upper gastrointestinal tract radiography ( Fig. 1C ). Blood tests showed only elevated lipase levels.

Initial medical management included the placement of a nasogastric tube, teaching postural adjustments, and initiating a high-calorie enteral diet. After a 6-day hospitalization, the patient was discharged home with continued dietary measures. Conservative treatment was primarily evaluated through regular follow-up consultations and clinical examinations by physicians and dietitians. Three weeks after medical management beginning, a follow-up upper gastrointestinal tract radiography showed a reduction in gastric and duodenal dilation, although some dilation persisted ( Fig. 1D ).

Despite this approach, the patient continued to experience pyrosis, epigastric pain, postprandial discomfort, and a need to split meals and lie on her left side after eating.

**Fig. 2:**
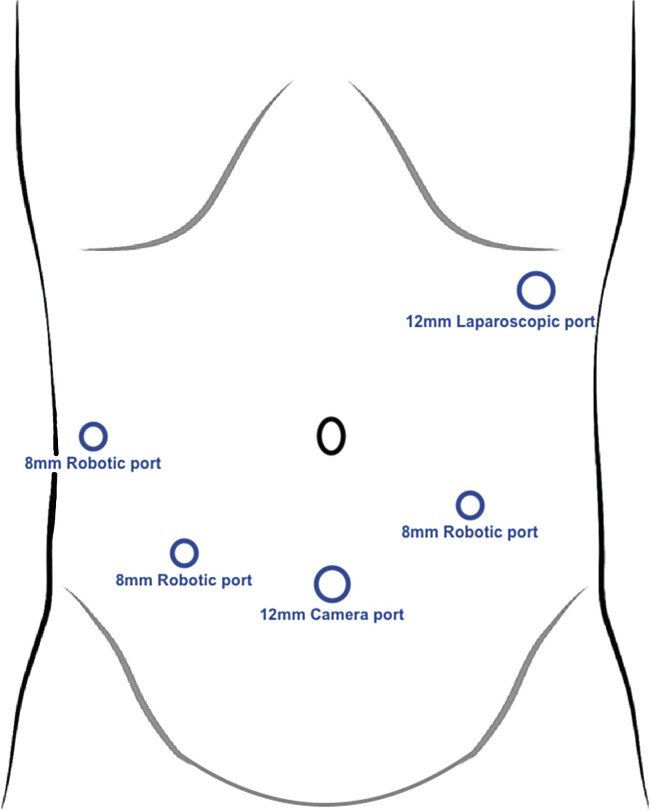
Trocar placement for robotic-assisted duodenojejunostomy without division of the fourth portion of the duodenum.

**Fig. 3:**
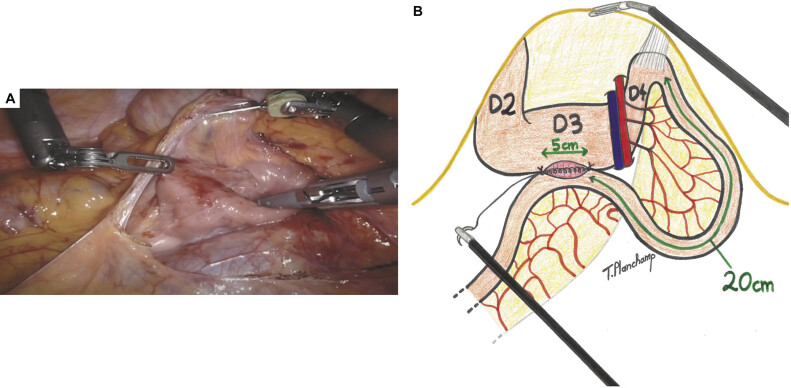
( **A** ) Intraoperative view of the opening of the transverse mesocolon and release of the adhesions of the posterior and third parts of the duodenum, allowing for easier mobilization. ( **B** ) At 20 cm from the Treitz angle, a jejunal loop was mobilized and fixed to the third portion of the duodenum using two stitches of Vicryl 4/0. The third portion of the duodenum and the antimesenteric side of the jejunum were then opened over 5 cm. A laterolateral duodenojejunal anastomosis was initiated with a posterior running suture of Vicryl 4/0, followed by an anterior running suture. D2, second portion of the duodenum; D3, third portion of the duodenum; D4, fourth portion of the duodenum.

As symptoms continued despite medical treatment, surgical intervention was decided upon 5 months after symptom onset, and a robotic-assisted duodenojejunostomy (DJ) without division of the fourth portion of the duodenum was performed. After trocar placement (four trocars for the robot and one for the assistant) ( Fig. 2 ), a transverse mesocolon opening was done ( Fig. 3A ). After a 5-cm opening of the third portion of the duodenum and the same opening on the antimesenteric side of the jejunum (20-cm downstream from the angle of Treitz), a laterolateral duodenojejunal anastomosis was performed, beginning with a posterior running suture using Vicryl 4/0, followed by an anterior one ( Fig. 3B ). A normal leak test was noted at the end of the procedure.

No postoperative complications occurred, and the patient experienced immediate relief from her symptoms and was discharged on postoperative day 4.

**Fig. 4:**
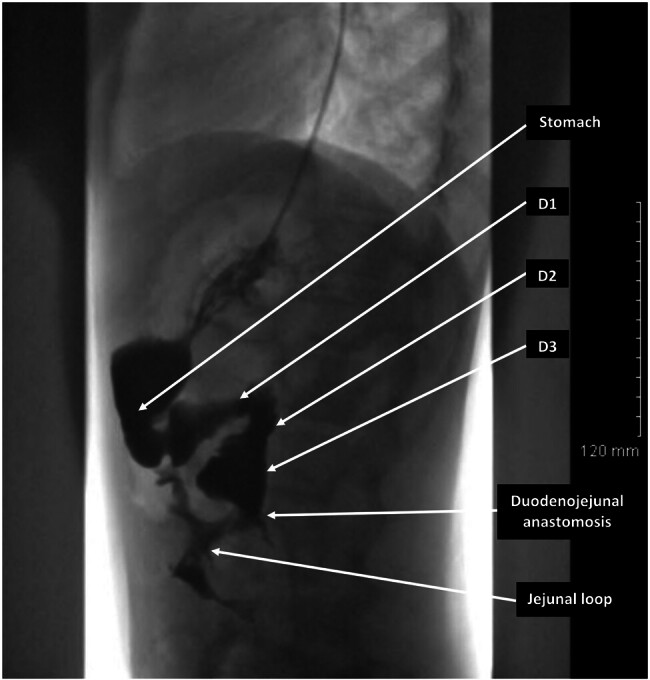
Lateral view of an upper gastrointestinal tract radiography performed 10 days after surgery, showing normal gastric and duodenal evacuation within a short time, with the persistence of slight distension in the first and second portions of the duodenum upstream of the anastomosis. D1, first portion of the duodenum; D2, second portion of the duodenum; D3, third portion of the duodenum.

An upper gastrointestinal radiography on postoperative day 10 showed normal gastric and duodenal evacuation within a short time, with however the persistence of slight distension in the first and second duodenum upstream of the anastomosis ( Fig. 4 ). No weight gain was observed during the first postoperative year.

No underlying cause was identified for her SMA syndrome, and she remains asymptomatic at the 7-year follow-up.

## Discussion

First described at autopsy by von Rokitansky in 1861, 3 SMA syndrome has a prevalence in the general population ranging from 0.013 to 0.3% and primarily affects females (ratio of 3:2), with a peak incidence between the ages of 10 and 40 years. 4

It is a life-threatening gastrovascular disorder characterized by compression of the third portion of the duodenum between the SMA and the abdominal aorta, resulting from a narrowing of the angle and a reduction in the fat space between these two structures.

Often a consequence of predisposing factors (anatomical abnormalities, genetic factors) associated with precipitating factors (reduction in mesenteric fat padding, acquired aorto-mesenteric angle closure), it may be idiopathic in 40% of cases. 5

An insidious onset of symptoms is found in 90% of cases and consists of postprandial epigastric pain and distension, abnormal bowel sounds on auscultation, early satiety, nausea, eructation, gastric reflux, bilious vomiting of large quantities of undigested food, and significant weight loss. Aggravated by eating and the supine position, symptoms can be relieved by the left lateral decubitus, prone, seated, or knee-to-chest positions, or by the Hayes maneuver. 6

An acute abdominal presentation with intestinal obstruction, as seen in our case, is rare but has been reported in a few previous studies. 7 8 9 Moreover, weight loss is not a necessary factor for the development of SMA syndrome in the pediatric population, 10 11 12 which is consistent with our case. Therefore, this condition can be difficult to consider when faced with nonspecific gastrointestinal symptoms, especially in cases of acute and idiopathic SMA syndrome like ours.

Diagnosis is made with a high level of clinical suspicion, followed by confirmatory imaging. Abdominal X-ray and upper gastrointestinal series confirm upper digestive obstruction. Fibroscopy shows gastroduodenal stasis, and an intravenous CT scan confirms a reduced angle (<22 degrees) between the SMA and the aorta, as well as a reduced aorto-mesenteric distance (<8 mm). 13 14 Moreover, various imaging modalities have recently been used to confirm SMA syndrome with good sensitivity, including abdominal ultrasound, magnetic resonance imaging, and endoscopic ultrasonography. 1

Conservative treatment is the first-line therapy and consists of gastroduodenal decompression with nasogastric tube suction, correction of hydroelectrolytic disorders, nutritional support with a dual high-calorie diet (enteral via a nasojejunal tube and parenteral), combined with postural adjustments (the optimal position varies from case to case, with the left lateral position effective in some cases, while the right lateral recumbent position and the sitting position may be more effective in others). 11 15 This treatment is effective in 71.3% of cases but is associated with a recurrence risk rate of 15.8%. 12

If conservative treatment fails, or if the patient's condition worsens and complications occur, surgical therapy must be considered. 1 There are currently no consensual guidelines about the best timing for transitioning to surgical options, but Shin and Kim suggest in their retrospective study that patients should have a trial of at least 4 to 6 weeks of conservative treatment with optimal nutritional support before considering surgery. 11

Over the years, various surgical techniques have been described to treat SMA syndrome, initially by laparotomy, then by laparoscopy, and now using a robotic approach. These techniques can be divided into diversion procedures (gastrojejunostomy, gastroduodenostomy, and DJ with or without division of the fourth portion of the duodenum) and anatomical modification procedures (Strong's surgery, anterior transposition of the third part of the duodenum, duodenal lowering, Ladd's procedure, and vascular transposition of the SMA). 1

With a success rate identical to that of open surgery, 16 associated with lower postoperative complications, the benefit of a laparoscopic approach is clear. 17

The most commonly advised surgical method is DJ, especially when performed laparoscopically, which has gradually become the standard surgical method because of its better success rate, lower incidence of blind loop syndrome, reduced morbidity, and shorter hospital stay, encouraging us to choose this technique in our case. 4 18 19 20 21 22 23

Only one case of robotically assisted DJ for SMA syndrome in children has been reported in the literature. It was performed in 2010 on an underweight 16-year-old girl with a BMI of 14.8. No complications were noted, and the patient was completely asymptomatic at postoperative month 21. 24 Like them, we found that robotic DJ appears to be a feasible technique for efficiently and safely managing SMA syndrome in the pediatric population, even in acute and idiopathic forms.

Surgery for SMA syndrome in children can benefit from robotic assistance in several ways. Indeed, children with SMA have a small abdominal cavity, which limits the use of intestinal staplers in a laparoscopic approach. In such cases, a laparoscopic manual anastomosis is required, which can be particularly challenging for surgeons with limited experience in laparoscopy, especially when performing complex suturing tasks such as DJ. 24 25

With its magnified view, enhanced maneuverability (seven degrees of freedom), and tremor correction, robotic assistance improves depth perception, facilitates access to confined surgical sites such as the ligament of Treitz, enhances dissection, and simplifies suturing. Moreover, robotic assistance is also beneficial for improving the economy of motion, which is particularly useful for complex procedures performed in limited workspaces, as is often the case in SMA syndrome. Last but not least, robotic assistance allows surgeons to operate in a more ergonomic position, reducing physical strain and fatigue, thereby enhancing both comfort and precision. 26 27

For these reasons, we believe that robotic assistance could be particularly beneficial for surgeons with limited experience in laparoscopic suturing, enabling them to perform complex procedures more efficiently and safely. Additionally, experienced surgeons may also benefit from robotic assistance, allowing them to work in a more ergonomically comfortable way. 27 28

## Conclusion

This case describes an acute and idiopathic form of SMA syndrome that required surgery. It highlights the necessity of considering this disease in cases of acute proximal bowel obstruction, even in atypical Wilkie's syndrome patients with a normal BMI. Indeed, idiopathic SMA syndrome represents the most challenging group of patients to diagnose, due to its nonspecific symptoms but is critical to diagnose early due to the positive response to treatment. DJ using a robotic approach has already been described in pediatric surgery and appears to be an effective and safe treatment for SMA syndrome in this population. Moreover, robotic assistance can be a valuable tool not only for surgeons with limited experience in laparoscopic suturing but also for experienced surgeons, allowing them to work in a more ergonomic manner.

## Notes

New Insights & the Importance for the Pediatric Surgeon The diagnosis of superior mesenteric artery syndrome can be challenging when faced with unspecific gastrointestinal symptoms suggestive of proximal bowel obstruction syndrome, especially in its acute and idiopathic form. Robotic duodenojejunostomy is an effective and safe treatment for superior mesenteric artery syndrome in pediatric surgery.
